# Comparison of Program Resources Required for Colonoscopy and Fecal Screening: Findings From 5 Years of the Colorectal Cancer Control Program

**DOI:** 10.5888/pcd16.180338

**Published:** 2019-04-25

**Authors:** Sujha Subramanian, Florence K.L. Tangka, Sonja Hoover, Maggie Cole-Beebe, Djenaba Joseph, Amy DeGroff

**Affiliations:** 1 RTI International, Waltham, Massachusetts; 2 Centers for Disease Control and Prevention, Atlanta, Georgia

## Abstract

**Introduction:**

Colonoscopy and guaiac fecal occult blood tests and fecal immunochemical tests (FOBT/FIT) are the most common colorectal cancer screening methods in the United States. However, information is limited on the program resources required over time to use these tests.

**Methods:**

We collected cost data from 29 Centers for Disease Control and Prevention Colorectal Cancer Control Program (CRCCP) grantees by using a standardized data collection instrument for 5 program years (2009–2014). We created a panel data set with 124 records and assessed differences by screening test used.

**Results:**

Forty-four percent of all programs (N = 124) offered colonoscopy (55 of 124), 32% (39 of 124) offered FOBT/FIT, and 24% (30 of 124) offered both. Overall, total cost per person was higher in program year 1 ($3,962), the beginning of CRCCP than in subsequent program years ($1,714). The cost per person was $3,153 for programs using colonoscopy and $1,291 for those using FOBT/FIT with diagnostic colonoscopy. The average clinical cost per person was $1,369 for colonoscopy and $280 for FOBT/FIT during the program (these do not reflect cost of repeated FOBT/FIT screens). Programs serving a large number of people had lower per-person costs than those serving a small volume, probably because of fixed costs related to nonclinical expenses.

**Conclusion:**

Colorectal cancer screening programs incur costs in addition to the clinical cost of the screening procedures to support planning and management, contracting with providers, and tracking patients. Because programs can achieve potential economies of scale, partnerships among smaller programs for screening delivery could decrease overall costs.

**Summary**

**What is already known on this topic?**

Three years of program data from the Centers for Disease Control and Prevention’s (CDC’s) Colorectal Cancer Control Program (CRCCP) showed that although the clinical cost of colonoscopy programs was higher than the clinical cost for guaiac fecal occult blood tests and fecal immunochemical tests programs, the cost of nonclinical services required to manage the programs and deliver the screenings was similar.

**What is added by this report?**

CDC and RTI International collected 5 years of cost data from 29 CRCCP grantees by using a standardized data collection instrument and assessed differences in costs by screening test used.

**What are the implications for public health practice?**

CRCCP grantees incurred costs in addition to the clinical cost of the screening procedures to support planning and management, contracting with providers, and tracking patients.

## Introduction

The Centers for Disease Control and Prevention (CDC) initiated the Colorectal Cancer Control Program (CRCCP) in 2009 to promote and provide screening to increase colorectal cancer (CRC) screening uptake in target populations. Under the program, CDC funded 29 grantees (25 states and 4 tribal organizations); grantees generally offered free screening colonoscopy or fecal tests to low-income people who were uninsured or underinsured. In an interim analysis of CRCCP, we assessed differences in costs of clinical and nonclinical screening incurred by CRCCP grantees during the first 3 years of the program and found that the cost of screening and diagnostic services per person served was $1,150 for colonoscopy programs and $304 for FIT/FOBT-based programs (1). Overall, FOBT/FIT-based programs and colonoscopy programs incurred substantial nonclinical costs per person served ($1,018 for colonoscopy and $980 for FIT/FOBT). Examples of nonclinical costs were managing contracts with providers and program management. These findings indicated that although the clinical cost of colonoscopy programs was higher than the clinical cost of FOBT/FIT programs, the cost of nonclinical services required to manage the programs and deliver the screenings was similar.

Our study expands on this prior analysis by evaluating cost over the 5-year period of the program and potential economies of scale in program implementation by assessing factors affecting the cost of screening provision. The large sample size available for analysis allowed us to perform multivariate analysis to evaluate the effect of large versus small programs on clinical and nonclinical costs, controlling for factors such as geographic location and type of screening test used. Prior research involving other screening programs indicated that these programs have high fixed costs (2–4). We theorized that programs that screen a large number of people may have a lower cost per-person than programs that screen a smaller number, which could have important implications for program planning and implementation.

## Methods

### Data collection

We developed a web-based cost assessment tool, the CRCCP Cost Assessment Tool (CRCCP-CAT), to collect information from CRCCP grantees on their program activities and expenditures. CRCCP-CAT is based on established methods for collecting cost data (5–8); a previously published article and a companion article in this collection describe the development and testing of CRCCP-CAT (9,10). For the CRCCP analyses, we collected data from each of the 29 CRCCP grantees. The grantees completed the web-based CAT annually, on the basis of program year, for a 5-year period beginning in July 2009 and ending in June 2014.

We collected cost information on the following: program funding source (CDC; other federal, nonfederal, state, or in-kind) and budget categories (staff salaries, contract expenditures, purchases of materials and equipment, and administration or overhead costs). Program staff members allocated these costs to screening activities, promotion activities, and overall program activities such as program management, partnership development, and administration. Promotion costs are discussed in a companion article in this collection (11). On the basis of the data provided in CRCCP-CAT, we allocated proportions of staff salary (based on number of hours and percentage of time worked) to specific activities. We then aggregated data on labor costs, nonlabor costs, and in-kind contributions for each activity for each grantee by year. Summaries of these data were sent to grantees annually for their review and approval.

The total sample size available for analysis was 124 program years over the 5-year period. We created a panel data set, which included each year of the program as 1 entry, and we reported our sample size in program years. Massachusetts (all years) and the Alaska Native Tribal Health Commission (all years) were excluded from the analyses because we were unable to disaggregate the clinical and nonclinical costs from contract payments in sufficient detail. Alabama, California, Iowa, New Mexico, and Oregon were all excluded in year 1 because they had not yet begun activities; Georgia, Michigan, and Nevada were not included in year 1 because they had not yet begun CRCCP. Georgia was also excluded from year 2 and Oregon from years 2 and 3 because their screening activities had not yet commenced during those years.

### Descriptive analyses

We stratified the programs by type of screening test used: colonoscopy, FOBT/FIT, and programs that used both tests. Fecal tests that include FOBT and FIT were offered as screening tests, and colonoscopy was offered as screening and for follow-up diagnostic procedures. Programs with both tests offered both fecal tests and colonoscopy for colorectal cancer screening. Some programs also offered surveillance colonoscopies, and these were reported separately from screening colonoscopies.

We identified key characteristics of the program, including the region and number of people served by the program, which was categorized as large (>500), medium (235–500), and small (<235) on the basis of the distribution of the underlying data. We also reported screening and diagnostic procedures for each type of program, including number of people who were screened or received surveillance colonoscopies, number of diagnostic procedures, and number of people identified with polyps. Use rates for the procedures were derived from information provided in CRCCP-CAT and from CRC clinical data elements that were collected from all programs by CDC (Office of Management and Budget [OMB] control no. 0920–0745).

We stratified cost information by the following activities: 1) direct clinical activities, such as provision of screening tests, diagnostic services, and surveillance procedures; 2) direct nonclinical activities, such as managing provider contracts and billing systems and providing patient navigation and patient support services; and 3) indirect nonclinical overarching activities, such as program management and administration (Box).

**Box. Component Activities of the Colorectal Cancer Control Program, 2009–2014**

**Direct clinical activities**

Screening and diagnostic services

Surveillance procedures

**Direct nonclinical activities**

Provider contracts, billing systems, other billing procedures

Patient navigation and support

Labor costs for screening and diagnostic services (if reported)

Ensure cancer treatment

Other screening provision activities

**Indirect nonclinical overarching activities (related to both screening promotion and screening procedures)**

Program management

Quality assurance/professional development

Partnership development and maintenance

Clinical and cost data collection and tracking

Program monitoring and evaluation

Administration

Other activities

We calculated the cost per person aggregated across all program years and the cost for each program year to examine patterns across the 5-year period. We estimated adjusted costs (multivariate regression controlling for region, size of population served, and type of screening test) for total cost per person for direct clinical costs, direct nonclinical costs, and indirect costs. We estimated the average incremental effect on cost of each explanatory variable as the difference from one of the exponentiated coefficients and multiplying by the mean of the variable. Cost data were adjusted for regional differences by using the Bureau of Labor Statistics’ Employment Cost Index.

### Multivariable regression specification

We used multivariate analysis to assess the effect of volume of people screened on cost per person. We examined the total cost per person served by 3 cost components: total direct clinical cost, total direct nonclinical cost, and total indirect cost (12–14). Results of a Hausman test indicated that a fixed effects model was not appropriate for this panel data and that a mixed effects model should be used (15). We used a generalized linear model (GLM) with log link and specified a gamma distribution. We included data for years 2 to 5 in the regression estimation. We excluded year 1 because this was the start-up period, anticipating that costs for this year would differ from other program years.

GLM with log link allowed us to exponentiate the coefficient estimates without the need for a retransformation as is required when estimating a log–linear model. Regression results were tabulated in terms of the incremental effect on average cost. We used the Stata statistical package, version 14.0 (StataCorp LLC) to conduct all regression analyses and statistical tests of the model.

## Results

Overall, 44.4% (55 of 124) of the programs assessed used colonoscopy as the primary screening test; 31.5% (n = 39) used FOBT/FIT, and 24.2% (n = 30) used both tests (Table 1). Of the programs that offered colonoscopy as the primary screening test, the greatest percentage (36.4%; n = 20) was in the Northeast, whereas of the 39 programs that offered FOBT/FIT as the primary screening test, most (61.5%; n = 24) were in the West. Forty-three percent (13 of 30) of the programs offering both tests were also located in the West. Programs offering FOBT/FIT and both types of tests were more likely to serve a large population (FOBT/FIT, 46.2% [18 of 39]; both tests, 63.3% [19 of 30]) than colonoscopy programs (10.9% [6 of 55]). On average, grantees using both tests screened 2,152 people over the 5-year period, followed by grantees using FOBT/FIT (683 people)) and grantees using colonoscopy (254 people). We also assessed program testing method by program characteristics (Table 2).

**Table 1:** Program Characteristics and Clinical Services by Type of Primary Screening Test for All Program Years^a^, Centers for Disease Control and Prevention Colorectal Cancer Control Program, 2009–2014

Characteristic	All (N = 124)	By Type of Test		
		Colonoscopy (n = 55)	FOBT/FIT (n = 39)	Colonoscopy and FOBT/FIT (n = 30)
**By screening test**	NA	44.4	31.5	24.2
**Region, mean (95% confidence interval)**				
Northeast^b^	20.2 (13.0–27.3)	36.4 (23.2–49.5)	0	16.7 (2.5–30.8)
Midwest^c^	18.6 (11.6–25.5)	9.1 (1.3–16.9)	28.2 (13.4–43.0)	23.3 (7.3–39.4)
South	17.7 (10.9–24.6)	23.6 (12.1–35.2)	10.3 (0.3–20.2)	16.7 (2.5–30.8)
West^c^	43.6 (34.7–52.4)	30.9 (18.3–43.5)	61.5 (45.6–77.5)	43.3 (24.5–62.2)
**Size of population screened by program size^d^, mean (95% confidence interval)**				
Large population^b^	34.7 (26.2–43.2)	10.9 (2.4–19.4)	46.2 (29.8–62.5)	63.3 (45.0–81.6)
Medium population^c^	36.3 (27.7–44.9)	45.5 (31.9–59.0)	38.5 (22.5–54.4)	16.7 (2.5–30.8)
Small population^b^	29.03 (20.93–37.13)	43.6 (30.1–57.2)	15.4 (3.5–27.2)	20.0 (4.8–35.2)
**Program reach, mean (95% confidence interval)**				
No. of people screened^b^	848.0 (540.8–1,155.3)	253.9 (208.6–299.2)	683.3 (518.5–848.1)	2151.5 (981.6–3321.4)
No. of people under surveillance^b^	23.5 (15.8–31.2)	15.7 (9.3–22.0)	21.3 (11.9–30.8)	40.8 (13.4–68.1)
No. of diagnostic tests performed^b^	41.3 (25.1–57.5)	7.3 (5.2–9.4)	44.2 (29.9–58.5)	99.9 (38.7–161.0)
No. of polyps detected^b^	47.5 (41.0–54.1)	61.4 (50.7–72.0)	27.2 (20.0–34.4)	48.6 (35.1–62.1)

Abbreviation: FOBT/FIT, fecal occult blood test/fecal immunochemical test; NA, not applicable.

a Unit of analysis is program year. Total sample size available for analysis was 124 program years over the 5-year period. We used the χ^2 ^test to test for differences across the types of colorectal cancer screening tests.

b *P* <.001.

c *P* <.05.

d Small population = 228,339–736,635; medium population = 854,624–1,618,255; large population = 1,749,719–9,472,316.

**Table 2:** Program Testing Method by Program Characteristics, Centers for Disease Control and Prevention Colorectal Cancer Control Program, 2009–2014^a^

Characteristic	Colonoscopy	FOBT/FIT	Colonoscopy and FOBT/FIT
**Region**			
Northeast (n = 20)	80.0 (60.8 to 99.2)	0	20.0 (7.9 to 39.2)
Midwest (n = 20)	20.0 (0.8 to 39.2)	50.0 (6.0 to 74.0)	30.0 (8.0 to 52.0)
South (n = 19)	57.9 (33.5 to 82.3)	21.15 (0.9 to 41.2)	21.1 (0.9 to 41.2)
West (n = 46)	30.4 (16.6 to 44.3)	47.8 (32.8 to 62.8)	21.7 (9.4 to 34.1)
**Population density**			
Large population (n = 38)	13.2 (1.9 to 24.4)	42.1 (25.7 to 58.6)	44.7 (28.2 to 61.3)
Medium population (n = 43)	NA	34.9 (20.0 to 49.7)	9.3 (0.3 to 18.4)
Small population (n = 24)	66.7 (46.3 to 87.0)	20.8 (3.3 to 38.4)	12.5 (−1.8 to 26.8)

Abbreviation: FOBT/FIT, fecal occult blood test/fecal immunochemical test; NA, not applicable.

a Values are percentage (95% confidence interval).

Overall, total cost per person decreased from year 1 ($3,962) to year 5 ($1,841); average cost across years 2,3,4, and 5 was $1,714. On average, the cost per person was highest in year 1 for each component. For example, in year 1, direct clinical cost per person was $1,068, decreasing in year 2 to $793, and remaining similar over the remaining years (Figure). Overall, the cost per person was high in year 1 compared with years 2 through year 5 for each component.

**Figure:**
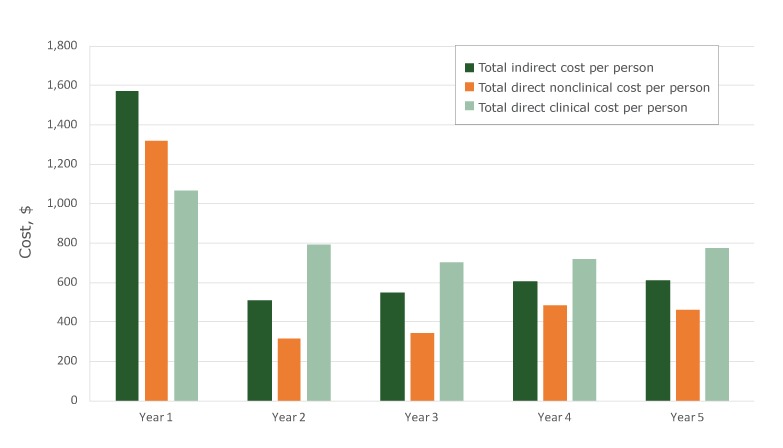
Five-year trends, cost per person screened, Colorectal Cancer Control Program, calculated on the basis of 124 program years, 2009–2014.

**Table:** 

Cost per Person, $	Year 1	Year 2	Year 3	Year 4	Year 5
Total indirect	1,573	510	546	605	61
Total direct nonclinical	1,321	313	34	484	458
Total direct clinical	1,068	793	702	717	772

Cost per person by type of screening test varied significantly across the 3 test types (Table 3). On average, screening tests cost $2,060 per person, ranging from $1,057 for both tests to $3,153 for colonoscopy. All components were, on average, most expensive for colonoscopy programs; total costs per person were $1,369 for direct clinical costs, $863 for nonclinical costs, and $921 for indirect costs. By comparison, total cost per person for FOBT/FIT were $280 for direct clinical costs, $375 for direct nonclinical costs, and $636 for indirect costs. Total per person costs for both tests were $411 for direct clinical costs, $173 for direct nonclinical cost was, and $473 for indirect cost.

**Table 3:** Cost per Person Screened by Type of Primary Test, Centers for Disease Control and Prevention Colorectal Cancer Control Program, 2009–2014

Type of Cost^a^	All	By Type of Test		
		Colonoscopy	FOBT/FIT	Colonoscopy and FOBT/FIT
Total cost per person^b^	2,060 (1,565–2,556)	3,153 (2,175–4,132)	1,291 (787–1,794)	1,057 (631–1,482)
Total direct clinical cost per person^b^	795 (631–958)	1,369 (1,069–1,669)	280 (216–343)	411 (283–539)
Total direct nonclinical cost per person^b^	543 (260–826)	863 (261–1,465)	375 (87–663)	173 (50–295)
Total indirect cost per person^b^	723 (535–912)	921 (552–1,290)	636 (390–882)	473 (238–708)

Abbreviation: FOBT/FIT, fecal occult blood test/fecal immunochemical test.

a All costs include in-kind contributions and were adjusted by using the Employment Cost Index for regional differences. Values are US dollars (95% confidence interval).

b *P* <.001. We used the χ^2 ^test to test for differences across the types of CRC screening tests.

Examining the estimates for adjusted total cost per person, we found that programs using colonoscopy screening had an average $1,104 higher total cost per person served compared with programs using FOBT/FIT tests in years 2 through 5 (Table 4). Increased size of the population served lowered total cost significantly; in years 2 through 5, average costs for programs with medium populations were $899 lower than programs with small populations served, and programs with large populations were $1,313 lower.

**Table 4:** Adjusted Cost per Person Screened, Years 2 to 5, Centers for Disease Control and Prevention Colorectal Cancer Control Program, 2009–2014^a^

Variable	Total Per Person	Direct Clinical	Direct Nonclinical	Indirect
**Region**				
South	1 [Reference]			
Northeast	(95) (−550 to 537)	(122) (−355 to 251)	70 (−177 to 587)	(179)^b^ (−301 to −2)
Midwest	(31) (−513 to 642)	28 (−274 to 524)	(6) (−218 to 447)	(76) (−231 to 150)
West	318 (−179 to 976)	(16) (−254 to 337)	222 (−77 to 797)	90 (−89 to 336)
**Size of population served by the program^c^**				
Small population served	1 [Reference]			
Large population served	−1,313^c ^(−1,412 to −1181)	−292^b^ (−445 to −62)	−352^d^ (−377 to −302)	−467^d^ (−495 to −429)
Medium population served	−899^d^ (−1,098 to −636)	−118 (−325 to 192)	−270^d^ (−333 to −150)	−320^d^ (−388 to −226)
**Screening test**				
FOBT/FIT	1 [Reference]			
Colonoscopy	1,104^d^ (439 to 1,974)	2,365^c^ (1 to 319 to 3 to 940)	76 (−139 to 469)	−64 (−196 to 115)
FOBT/FIT and colonoscopy	−215 (−563 to 237)	249 (−49 to 675)	−108 (−245 to 151)	−139 (−252 to 15)

Abbreviation: FOBT/FIT, fecal occult blood test/fecal immunochemical test.

a All costs include in-kind contributions and were adjusted by using the Employment Cost Index for regional differences. All estimates are based on multivariate analysis; each column is a separate regression. Values are dollars (95% confidence interval). Results are for years 2–5 (N = 105).

b *P* <.05.

c Small population = <235; medium population = 235–500; large population = >500.

d *P* <.001.

The total number of people screened had some effect on the direct clinical cost per person; programs with large populations screened had $292 lower costs than programs with small populations screened. Colonoscopy programs had a higher direct clinical cost than FOBT/FIT programs ($2,365 higher).

Our estimates for total direct nonclinical cost per person served show that type of screening test did not affect direct nonclinical costs (Table 4). Similar to total costs, costs for programs with large populations served were $352 lower than programs with small populations served, whereas costs for programs with medium populations served were $270 lower.

We also found that total indirect cost per person served was significantly lower among programs with larger populations served (Table 4). The average indirect cost per person served was $467 lower among programs with a large population served and $320 lower among programs with a medium population served, compared with programs with a small population served. Region also significantly affected these costs. Programs in the Northeast had an average $179 lower indirect cost per person served than programs in the South.

## Discussion

We compared the clinical and nonclinical costs across program years among CRCCP grantees offering colonoscopy, FOBT/FIT, or both tests for CRC screening. Our findings expand on our prior analysis and use 5 years of data to quantify the presence of economies of scale — programs that screen a larger number of people had lower cost per person than programs that screen a smaller number of people. After controlling for type of screening test, programs serving large and medium-size populations had per-person costs that were about $1,300 and $900 lower, respectively, than programs serving small populations.

Another key finding from our study was that public health–led CRCCP programs incurred substantial nonclinical costs. These costs are important to consider when planning future programs. On average, these costs were lower for programs with large patient volumes than for programs with small patient volumes. These findings indicate that substantial fixed costs are associated with nonclinical activities. These results are further evidence that economies of scale exist in CRC screening programs, as reported in other studies (2–4).

Analysis of patterns in cost per person indicated differences in cost between the first year and subsequent years of the program. The average cost per person served in the first year was twice that of the other years. This higher cost in the first year likely reflects start-up costs incurred by the programs while planning and beginning implementation. Furthermore, the number of people screened was generally lower in the first year. Any nonclinical costs incurred in the first year would have to be distributed across a much smaller cohort. High start-up costs in the initial years of the program were also reported in other studies (3,16,17), suggesting that first-year costs should perhaps be analyzed separately and not pooled with costs incurred in subsequent program years.

Additionally, we identified some differences across programs related to type of screening test used. The clinical cost of colonoscopy was almost 5 times the cost of FOBT/FIT per person when screening and diagnostic follow-up tests were included. Therefore, programs that use colonoscopy will only be able to screen about one-fifth the number of people that FOBT/FIT programs can for the same level of funding in the initial years of the program. This cost would only affect the number of people screened in the short term because colonoscopy is recommended every 10 years for those at average risk and with normal results, whereas FOBT/FIT is recommended to be performed annually. The clinical costs over a 10-year period for colonoscopy and FOBT/FIT may not be substantially different. We did not find any consistent evidence of variation in indirect costs and direct nonclinical costs by type of screening test used. FOBT/FIT tests were the preferred approach when the primary goal was to offer first-time screening to a large cohort over a short period; we did not study FOBT/FIT with repeated testing. Future studies could assess additional program costs that may be incurred, to ensure adherence with colorectal cancer screening recommendations over the long term. Furthermore, we found some regional and screening test–related differences in indirect costs; future studies could explore whether these findings are replicated in other settings and the possible reasons for these differences.

The strength of the present cost analysis is that we were able to perform high-quality analysis by collecting and quantifying resources and using consistent definitions for program activities. Furthermore, we collected data across 5 years from multiple programs to yield a substantial panel data set of 124 program years. These cost data were consistently collected over a longer period than any other federally supported screening program and allowed for multivariate analysis, controlling for some determinants of potential variation across the programs.

Our analysis has several potential limitations. First, we used program year to assess potential year-to-year variation, but programs generally operate on a continuous basis. Therefore, screening tests could be performed in one year, while diagnostic follow-up and treatment, if required, could be provided in the following year. As a result, classification of costs and number screened in specific periods are not always an accurate reflection of program activities. Second, the study does not account for cost per patient over an extended period to compare the long-term cost of colonoscopy versus FOBT/FIT-based programs. We only report cost for the first testing period (screening and diagnostic tests required), and our estimates do not provide the overall cost of FIT/FOBT and colonoscopy programs. Third, there could be variation across programs by type of screening test used (eg, colonoscopy vs FOBT/FIT). This variation could influence the costs reported and may not have been adequately controlled in our analysis. Future research could systematically assess the factors that can lead to cost differences of activities by type of screening test selected.

Our analysis of the activity-based cost data across 5 years of the CRCCP reveals potential economies of scale: programs with larger screening volume incurred a lower cost per person served than smaller-volume programs. Therefore, encouraging partnerships to foster large-scale programs could be more efficient than funding multiple small screening programs. Additionally, CRC screening programs incur substantial nonclinical costs, regardless of type of test the program offers. Future CRC control programs might consider both these clinical and nonclinical costs when planning program implementation and evaluating program cost-effectiveness.

## References

[1] Subramanian S , Tangka FKL , Hoover S , Royalty J , DeGroff A , Joseph D . Costs of colorectal cancer screening provision in CDC’s Colorectal Cancer Control Program: comparisons of colonoscopy and FOBT/FIT based screening. Eval Program Plann 2017;62:73–80. 10.1016/j.evalprogplan.2017.02.007 28190597 PMC5863533

[2] Subramanian S , Ekwueme D , Gardner J , Kramer C , Bapat BS , Tangka F . Identifying and controlling for program-level differences in comparative cost analysis: lessons from the economic evaluation of the National Breast and Cervical Cancer Early Detection Program. Eval Program Plann 2008;31(2):136–44. 10.1016/j.evalprogplan.2008.02.002 18359084

[3] Ekwueme DU , Gardner JG , Subramanian S , Tangka FK , Bapat B , Richardson LC . Cost analysis of the National Breast and Cervical Cancer Early Detection Program — selected states 2003 to 2004. Cancer 2008;112(3):626–35. 10.1002/cncr.23207 18157831

[4] Trogdon JG , Ekwueme DU , Subramanian S , Crouse W . Economies of scale in federally-funded state-organized public health programs: results from the National Breast and Cervical Cancer Early Detection Programs. Health Care Manage Sci 2014;17(4):321–30. 10.1007/s10729-013-9261-z 24326873 PMC5840803

[5] Anderson DW , Bowland BJ , Cartwright WS , Bassin G . Service-level costing of drug abuse treatment. J Subst Abuse Treat 1998;15(3):201–11. 10.1016/S0740-5472(97)00189-X 9633032

[6] Drummond M , Schulpher M , Torrance G , O’Brien B , Stoddard G . Methods for the economic evaluation of health care programmes. Oxford (UK): Oxford University Publishing; 2005.

[7] French MT , Dunlap LJ , Zarkin GA , McGeary KA , McLellan AT . A structured instrument for estimating the economic cost of drug abuse treatment. The Drug Abuse Treatment Cost Analysis Program (DATCAP). J Subst Abuse Treat 1997;14(5):445–55. 10.1016/S0740-5472(97)00132-3 9437614

[8] Salomé HJ , French MT , Miller M , McLellan AT . Estimating the client costs of addiction treatment: first findings from the client drug abuse treatment cost analysis program (Client DATCAP). Drug Alcohol Depend 2003;71(2):195–206. 10.1016/S0376-8716(03)00133-9 12927658

[9] Subramanian S , Ekwueme DU , Gardner JG , Trogdon J . Developing and testing a cost-assessment tool for cancer screening programs. Am J Prev Med 2009;37(3):242–7. 10.1016/j.amepre.2009.06.002 19666160

[10] Hoover S , Subramanian S , Tangka F . Developing a Web-Based Cost Assessment Tool for Colorectal Cancer Screening Programs. Prev Chronic Dis 2019;16:180336. 10.5888/pcd16.180336 PMC6513486 31050637

[11] Tangka F , Subramanian S , Hoover S , Cole-Beebe M , DeGroff A , Joseph D , Cost and factors associated with expenditures on screening promotion activities in CDC’s Colorectal Cancer Control Program. Prev Chronic Dis 2019;16:180337. . Forthcoming. 10.5888/pcd16.180337 PMC6583814 31172915

[12] Liu L , Strawderman RL , Cowen ME , Shih Y-CT . A flexible two-part random effects model for correlated medical costs. J Health Econ 2010;29(1):110–23. 10.1016/j.jhealeco.2009.11.010 20015560 PMC2824028

[13] Manning WG , Basu A , Mullahy J . Generalized modeling approaches to risk adjustment of skewed outcomes data. J Health Econ 2005;24(3):465–88. 10.1016/j.jhealeco.2004.09.011 15811539

[14] McCulloch CE . Maximum likelihood algorithms for generalized linear mixed models. J Am Stat Assoc 1997;92(437):162–70. 10.1080/01621459.1997.10473613

[15] Griswold M , Parmigiani E , Potoksky A , Lipscomb J . Analyzing health care costs: a comparison of statistical methods motivated by Medicare colorectal cancer charges. Biostatistics 2004;1(1):1–23. 14744824

[16] Tangka FK , Subramanian S , Bapat B , Seeff LC , DeGroff A , Gardner J , Cost of starting colorectal cancer screening programs: results from five federally funded demonstration programs. Prev Chronic Dis 2008;5(2):A47. 18341782 PMC2396978

[17] Subramanian S , Tangka FK , Hoover S , Degroff A , Royalty J , Seeff LC . Clinical and programmatic costs of implementing colorectal cancer screening: evaluation of five programs. Eval Program Plann 2011;34(2):147–53. 10.1016/j.evalprogplan.2010.09.005 21036399

